# Editorial: Biostimulants in agriculture II: towards a sustainable future

**DOI:** 10.3389/fpls.2024.1427283

**Published:** 2024-05-31

**Authors:** Maurizio Ruzzi, Giuseppe Colla, Youssef Rouphael

**Affiliations:** ^1^ Department for Innovation in Biological, Agri-food and Forestry Systems, University of Tuscia, Viterbo, Italy; ^2^ Department of Agriculture and Forest Sciences, University of Tuscia, Viterbo, Italy; ^3^ Department of Agricultural Sciences, University of Naples Federico II, Portici, Italy

**Keywords:** non-microbial biostimulants, microbial biostimulants, European Regulation 2019/1009, abiotic stresses, nutrient use efficiency, quality

## 1 Introduction

Current socio-political scenarios are pushing the agricultural sector towards a rapid ecological transition. For this reason, modern agriculture is forced to expand its ordinary practices to achieve greater sustainability in line with the principles of economic and environmental circularity (Velasco-Muñoz et al., 2021). Nonetheless, current challenges such as the need to increase productivity and the efficiency of non-renewable resources in increasingly unfavorable contexts strain agro-ecosystems resilience. Over the last century, synthetic agrochemicals have been the only tool capable of increasing yields and ensuring satisfactory production volumes throughout the seasons (Donate and Frederico, 2019). Awareness of the weighty environmental impact associated with the disproportionate use of these chemicals has stimulated research and development of technological innovations capable of improving the sustainability of agricultural production without compromising yield and quality. In recent decades, biostimulants have represented an efficient and virtuous innovation capable of improving tolerance against a wide range of stressors, stimulating the growth and productivity of crops while efficiently utilizing nutrients (nutrient use efficiency). According to the latest European Regulation 2019/1009, plant biostimulants are defined as “*a product that stimulates the nutritional processes of plants independently of the nutrients it contains, with the sole aim of improving one or more of the following characteristics of plants or their rhizosphere: (1) the efficiency of nutrient use; (2) tolerance to abiotic stress; (3) qualitative characteristics; (4) the availability of nutrients confined in the soil or rhizosphere.*” Beneficial microorganisms and numerous natural substances and derivatives of natural and/or synthetic compounds are considered plant biostimulants; these include (1) humic substances, (2) protein hydrolysates and amino-acid based products, (3) macro- and micro-algae extracts, and plant extracts, (4) silicon; (5) Arbuscular Mycorrhizal Fungi (AMF); and (6) plant growth-promoting rhizobacteria (PGPR) belonging to the genera *Azotobacter*, *Azospirillum* and *Rhizobium* spp (EU, 2019). Although biostimulants were initially designed as a helpful tool mainly in organic farming, they are now used in all cropping systems (organic, conventional, and integrated) in the open field and protected cultivation.

Driven by the strong dependence on the agricultural sector for food, the economic size of the biostimulants market reached USD 3 billion in 2022 and is expected to grow at a compound annual growth rate (CAGR) of 10.5% over the next decade. Europe dominated the market with a revenue share of over 37.0% (GVR, 2023). This is partly attributed to the support regulations to stimulate organic food production, which are expected to drive the market in Europe and beyond in the future. The strong interest in the topic of biostimulants is confirmed by the data available on Scopus (www.scopus.com). Taking the last ten years (2013–2023) as a reference, searching for the term *‘plant biostimulants*’ more than 2000 scientific papers were found. These numbers are not surprising since, as reported by several authors (Jindo et al., Tripathi et al., Herrmann et al., and Li et al.), the use of substances and microorganisms with biostimulant action is of interest in all productive agricultural systems (horticulture, arboriculture and viticulture, cereal cultivation and industrial crops), even if their efficacy as underlined by the same authors varies depending on the cultivation conditions and the sensitivity of the reference species. The present topic brings together 77 scientific contributions (68 research articles and 9 reviews; **Supplementary Table 1**) investigating both under optimal and sub-optimal conditions, the productive and/or qualitative response of crops of agronomic interest to the application of biostimulants, as well as the use of compounds with elicitor action.

## 2 Biostimulants improve tolerance to abiotic stress

Due to the unpredictable and damaging effects of climatic anomalies associated with climate change, crops experience many stresses that inevitably reduce yield and final product quality, thus making it difficult to achieve food security objectives (Goud et al., 2022). Using products with biostimulant action represents one of the most innovative and promising tools to counter these stressors and reduce the gap between achievable yield and potential yield (Rouphael and Colla, 2020). Considering that plants respond to environmental stresses through adaptive cellular reprogramming, the work of Bharadwaj et al. emphasizes that during the delicate phase of seed germination, cellular sucrose levels are a very important sensor of environmental stresses that can be used to improve germination and disease detection. As discussed by Atta-Boateng and Berlyn, yield reductions are not exclusively attributable to specific stress but to a co-presence of them. Choosing cowpea (*Vigna unguiculata* (L.) Walp.), adapted to nitrogen dependence, as a model crop, Atta-Boateng and Berlyn. observed that the use of non-hormonal biostimulants (product based on antioxidants and amino acids prepared by the laboratory of the Yale School of the Environment) is an efficient tool to promote productivity when it is limited by osmotic stresses attributable to high temperatures, water limitations, and sub-optimal moisture conditions. Ambrosini et al. evaluated the ability of an animal-derived protein hydrolysate to mitigate the incidence of drought stress and root oxygen and iron deficiency conditions in hydroponically grown maize plants (*Zea mays* L.). The same authors also emphasized once again how the positive effects cannot be attributed to an exclusive mechanism of action but are instead the result of the commercial formulation’s synergistic action of different components (mainly peptides). In this context, Antonucci et al., using a mixed phenotypic-omics approach, observed the mechanisms implemented following the use of a commercial glycine betaine-based biostimulant in tomato (*Solanum lycopersicum* L.) plants (during the flowering phase) subjected to water stress. The authors reported a priming effect of the biostimulant aimed at stabilizing the photosynthetic machinery, both mediated by the hyperaccumulation of carotenoids and lipids and improved water use efficiency. After foliar application of melatonin (100 μM), Zhao et al. observed an improvement in the growth and photosynthetic capacity of maize plants grown under drought conditions attributable to increased proline levels. In addition, the application of melatonin increased enzyme activities involved in nitrogen metabolism. Hernandiz et al., after optimizing the dose of putrescine (Put) and spermidine (Spd) in a growth chamber experiment, evaluated the effect of these two polyamines on maize plants grown in a protected environment under optimal irrigation and deficit irrigation conditions. Using Put and Spd with a higher meal’s mineral content (Ca, Cu, and Zn) improved the saleable yield under optimal and sub-optimal conditions. Starting from the assumption that continuous states of water stress (even mild ones) negatively affect plant germination, development, and proper growth, Hameed et al. evaluated on wheat (*Triticum aestivum* L.) the effect of sodium silicate (SS) as a priming agent to reduce the effects of water stress (50% of soil water retention capacity). At plant maturity, the authors observed, under water-limited conditions, an improvement in yield parameters induced by the positive action of Si for plants previously primed with SS. Also, on wheat, Lalarukh et al. investigated the efficacy of the single or combined use of *Pseudomonas aeruginosa* (Pa) (10^8^ CFU ml^–1^) and moringa leaf extracts at different doses (MLE 1 = 1:15 v/v and MLE 2 = 1:30 v/v) under drought and nutritional stress conditions. At the end of their field trial, the authors observed for plants treated with Pa + MLE 2 a significant improvement in yield traits in stressed wheat plants. The synergy between microbial and non-microbial biostimulants is paving the way for research into biopreparations that can further improve yield performance. Considering this, Kang et al. summarized in their review the potential synergistic effects of algae and bacteria on plant production as biostimulants and biofertilizers. In this context, a commercial biostimulant based on glycine betaine (GB), distributed at a rate of 6 kg ha^–1^, was tested. The trials were conducted on tomato plants during the flowering phase in the greenhouse. A factorial combination of two irrigation (water stress and well-watered) and two biostimulant treatments (treated and control) was adopted, along with a mixed phenotypic-omics approach. The authors demonstrated the priming effect of the biostimulant on drought tolerance, detoxification, and stabilization of the photosynthetic machinery. The metabolic profile and photosynthetic performance results suggest increased effective water utilization through lipid hyperaccumulation and leaf thickening. Also, on tomatoes subjected to combined water and nutrient deficiency, Kalozoumis et al. evaluated the possible effects of inoculating plant growth-promoting rhizobacteria (PGPR) with the agronomic practice of grafting. At the end of the experimental study, the authors emphasized the importance of the combined selection of PGPR strains and rootstock genotypes to achieve a substantial benefit under combined stress conditions. In a similar study, Toubali et al. evaluated the single and/or combined effects of inoculating arbuscular mycorrhizal fungi and compost on the field production of quinoa (*Chenopodium quinoa* Willd.) under water stress. Microbial biostimulation and the presence of compost ensured that plants subjected to suboptimal water levels maintained high production yields due to specific morpho-physiological adaptations. The review by Etesami et al. investigates in detail the contribution of AMF, phosphate-solubilizing bacteria (PSB), and silica in improving the availability and efficacy of phosphate, a limestone-limiting macroelement, especially in calcareous and acidic soils. Wang et al. observed a significant increase in maize Bt yield, foliar contents of salicylic acid (SA) and jasmonic acid, Bt toxin content, and gene expression after inoculation of an AMF species (*Glomus caledonium*). However, even though mutualistic relationships between mycorrhizal fungi and plants are routinely ‘exploited’ to improve the latter’s tolerance to a multitude of abiotic and other stresses, most of the scientific literature is mainly concerned with the study of arbuscular mycorrhizae. The review proposed by Wei et al. focused on describing the positive effects (increased tolerance to drought, heavy metals, soil salinity, and pathogen infections) of ericoid mycorrhizae on plants belonging to the *Ericaceae* family. Nazari et al. evaluated the interactive effects between plant and bacteriocin molecules to reduce the negative impact of low-temperature stress (5°C) on canola. Specifically, treatment with 10^–9^ thuricin 17 (Th17) significantly improved seed vigor index, germination rate, root and shoot length, and fresh weight of seeds under optimal, and low-temperature conditions. According to the study by Sun et al., the definition and characterization of vegetative attributes through leaf spectral indices could provide useful information to better understand plant-microbe relationships by optimizing bioproducts. The research by Melini et al. emphasizes that the use of selected strains of *Pantoea agglomerans* can be a promising approach for the development of new plant biostimulants for agricultural use through the fermentative production of auxin/indole 3-acetate (IAA). Similarly, through *in vitro* phenotypic analyses, Gutierrez-Albanchez et al. demonstrated the ability of a new *Pseudomonas*, designated as strain BBB001T, to produce auxins and siderophores. Subsequently, the same authors confirmed the potential of this strain by testing on olives (*Olea europaea* L.) and tomatoes (*S. lycopersicum* L.) grown under water limitation or iron deficiency, respectively. Based on the phosphate-solubilising capacity and the production of IAA, *Pseudomonas* sp. strain 1008 (isolated from the rhizosphere of wheat plants), Díaz et al. observed an increase in grain yield after inoculation of the biostimulant bacterium on wheat seeds from 2010–2017. Further genome sequencing analyses revealed the ability of strain 1008 to efficiently utilise organic and inorganic sources of phosphorus and compete for iron scavenging as well as the production of auxins and GABA. To limit the negative effects induced by iron deficiency on *Cinnamomum camphora*, Kong et al. investigated the role of *Rahnella aquatilis* (JZ-GX1) by assessing the active iron content, chlorophyll, and effects on the microbial community. The results showed that inoculation of JZ-GX1 increased chlorophyll content and enzyme activities (SOD, POD, CAT, and APX) on stressed plants. On the other hand, Macias-Benitez et al. investigated the biostimulating capacity of a rice bran enzyme extract (RBEE) used as a protective agent against oxidative damage from ozone exposure in the growth chamber. Pepper plants (*Capsicum annum* L.) treated with RBEE showed a reduction in photosynthetic limitation attributable to the oxidative action of ozone. Pot experiments conducted by Raza et al. showed that applying a vitamin B complex significantly improved the photosynthetic performance of radish plants, underlining the positive contribution of natural biostimulant products on crop productivity. Although most metals are essential trace elements for plants, their excess (often caused by human activities) is responsible for damaging effects that limit plant growth and metabolism. Considering this, the use of biostimulants to improve the effectiveness of ordinary remediation practices has been attracting increasing attention in recent years. Exploiting the synergistic effect between silicon nanoparticles and iron oxide nanoparticles, Koleva et al. observed on *Phaseolus vulgaris* plants grown on cadmium (Cd)-contaminated soil an improvement in growth and a significant reduction in electrolyte loss and malondialdehyde (MDA) content. Similarly, the foliar application of liquiritoside (liquiritin), a flavonoid complex isolated from *Glycyrrhizae radix*, proposed by Akram et al. on Chinese cabbage (*Brassica rapa* subsp. *parachinensis*) mitigated the phytotoxic effects induced by the presence of lead (Pb) through a reduction in the MDA, H_2_O, and cysteine content of the treated plants. Naveed et al. evaluated the potential of compost, biochar, and co-composted biochar on *Brassica napus* plants grown on chromium (Cr)-contaminated soil. At the end of the experiment, Naveed et al. observed a significant improvement in biometric and physiological traits of Cr-stressed plants after using co-composted biochar. Mustafa et al., in a pot experiment, compared the effects of biochar obtained from agricultural waste (AB) and food waste (FWB) on key indicators of soil microbiological health with and without mineral fertilizer inputs. The results showed changes in the chemical properties of the soil after fertilization with AB and FWB, which stimulated the growth of lettuce in the growth chamber. Similarly, Hammerschmiedt et al. studied the impact of different digestates (applied at a rate of 40 t ha^–1^) on soil chemistry and lettuce yield. At the end of the experiments, the authors observed a positive stimulation of soil microbial activity following the use of legume digestates, which could, however, accelerate nitrogen mineralization processes.

One of the environmental factors that most reduces crop growth is undoubtedly salt stress (Abdelhamid et al., 2020). Intense evapotranspiration activity stimulated by environmental warming combined with poor irrigation (often with water of poor quality) increasingly causes secondary soil salinization problems. Gao et al. evaluated the potential beneficial effects of 2-keto-L-gulonic acid (2KGA) on Chinese cabbage (*Brassica campestris* ssp. *chinensis* L.) subjected to salt stress (100 mM NaCl). The results suggest that applying 2KGA by promoting the synthesis of L-ascorbic acid can effectively alleviate the inhibitory and phytotoxic effects of NaCl salinity. Exogenous application of 5-aminolevulinic acid significantly reduced damage caused by moderate NaCl stress (50 mmol L^–1^) on cucumber seedlings due to increased root cytoactivity and improved Na^+^ redistribution and compartmentalization (Wu et al.).

On the other hand, Sorrentino et al. observed after the application on tomato and lettuce (grown under fully controlled conditions) of a plant biostimulant based on plant-derived protein hydrolysate how the positive action of the tested product induced species-specific adaptive responses that helped to alleviate the harmful effects of NaCl salinity. Similarly, Zuluaga et al. evaluated the different sensitivity of lettuce and tomato plants to high salinity conditions (80 mM for lettuce and 120 mM for tomato) and the possible protective effects of 4 different plant-derived protein hydrolysates (C: *Malvaceae*-derived, P: *Poaceae*-derived, D: legume-derived commercial ‘Trainer^®^’ and H: legume-derived commercial ‘Vegamin^®^’). Although lettuce had a constitutive higher sensitivity to salinity than tomato, the efficacy of biostimulants was more evident in lettuce. Regarding the positive role played by microbial biostimulants, Mellidou et al. observed on tomato plants subjected to salt stress (200 mM NaCl) an up-regulation of secondary metabolism after inoculation with *Pseudomonas oryzihabitans*, which enabled the salinized plants to respond more readily to salt-induced extremes. Also, on tomatoes, Becerril-Espinosa et al. observed how establishing an endophytic interaction with the actinobacterium *Salinispora arenicola* promotes germination and growth of roots and shoots under saline and non-saline conditions. An *in-vitro* study by Naamala et al. evaluated the stimulatory effect of cell-free supernatant (CFS) obtained from *Lactobacillus helveticus* EL2006H at different concentrations (0.2 and 1.0% *v/v*) on the germination and biometric traits of maize and soybean grown with NaCl levels of 100 and 150 mM. Although the results showed an efficacy of CFS, these were strongly influenced by the different concentrations used and the NaCl levels and plant species evaluated. In a hydroponic experiment, Tarroum et al. observed on tobacco plants an improved salt tolerance (250 mM) following the application of a nutrient solution of a cell-free supernatant obtained from a halotolerant fungal strain (A3) identified as *Penicillium olsonii*. The results obtained by Xiao et al. indicated that the application of *Bacillus cereus* G2 promoted the growth of *Glycyrrhiza uralensis* (Fisch. ex DC.) plants subjected to salt stress (75 mM NaCl) due to a significant improvement in photosynthetic efficiency and an increased in carbohydrate products from carbohydrate transformation. At different salinity levels (0, 20, 50, and 75 mM), Mhada et al. observed mitigation of the negative impact of salt stress on *Phaseolus vulgaris* plants previously primed and/or coated with a mixture of *Rhizobium tropici* and trehalose. Christakis et al. first identified endophytic bacteria of three aliphatic species: *Cakile maritima*, *Matthiola tricuspidate*, and *Crithmum maritimum*. Subsequently, they observed their ability to grow at high NaCl levels and inhibit the development of phytopathogens such as *Verticillium dahliae*, *Ralstonia solanacearum*, and *Clavibacter michiganensis*. Based on the results, the authors suggest testing halophyte endophytes as new ‘bio-inoculant’ agents to improve crop response to biotic and abiotic (salinity) stress conditions. Zhang et al., after identifying and isolating a new endophytic bacterium designated as *Bacillus altitudinis* strain using 16S rDNA sequencing, evaluated through bioassays the positive influence of this on the growth and development of various plant species.

## 3 Biostimulants increase tolerance to biotic stress

The possibility of reducing agrochemicals by developing bio-control agents would help the entire agricultural sector become more free of dependence on conventional chemical inputs, offering sustainable solutions to producers and consumers (Lahlali et al., 2022). Zhang et al., after isolating 60 actinomycetes, observed significant antifungal activity on 17 of them against *Fusarium oxysporum* f. sp. cubense tropical race 4 (TR4), a pathogen that causes one of the most common soil diseases globally. Xu et al. reported that in cucumber plants, there was a strong biocontrol activity against *F. oxysporum* sp. *cucumerinum* by a newly isolated *Bacillus subtilis* strain named YB-04. This effect was attributed to the ability of this strain to secrete amylase, cellulose, and extracellular protease, as well as produce indoleacetic acid and siderophores. After evaluating *in vitro*, the antagonistic activity of *Bacillus tequilensis* GYUN-300 (GYUN-300) against the fungal pathogen *Colletotrichum acutatum*, Kwon et al. observed on red pepper plants inoculated with GYUN-300 a reduction in the incidence of *Colletotrichum acutatum* attacks compared to untreated controls. Drobek et al. confirmed the efficacy of several microbial consortia consisting of bacteria such as: AF117AB (*Paenibacillus polymyxa*), Sp115AD (*Bacillus subtilis*), AF75AB2, Sp115AD, AF75BC (*Bacillus* sp.), AF75AA, AF75AD (*Streptomyces* sp.), JAFGU (*Lysobacter* sp.) and AF70AC (*Pseudomonas* sp.), in controlling the main microbial pathogens *(Botrytis cinerea*, *Verticillium* sp., *Phytophthora* sp. and *Colletotrichum* sp.) of strawberries, also underlining the positive impact on the final fruit quality. Seeds of two different rice varieties coated with *Trichoderma* strains inhibited the incidence of pathogens. They improved the chlorophyll content and vigor of the treated plants (Swain et al.). Valverde et al. evaluated the antifungal activity of 20 commercial macroalgae products against two post-harvest pathogens (*B. cinerea* and *Penicillium digitatum*) of fruit. Following the chemical evaluation of the tested products, the results confirmed that some specific bioactive components reduced the incidence of *B. cinerea.* At the same time, the control of *P. digitatum* was attributed to the presence of carbohydrates and polysaccharides. The application of a consortium of *Trichoderma asperellum* GDFS1009 and *Beauveria bassiana* OFDH1–5 on maize plants not only increased antioxidant enzyme activities and chlorophyll content but also decreased the survival of *Ostrinia furnacalis* (Batool et al.).

## 4 Biostimulants increase nutrient use efficiency

To maximize crop yields, farmers rely on large quantities of chemical fertilizers that are not always efficiently absorbed by the plant. In addition, their industrial production has an environmental impact that is no longer sustainable. Incentivizing natural products that affect N and P utilization is necessary for present and future agriculture (Umar et al., 2020). As argued by Reis et al., among the most sustainable agricultural techniques, the use of phosphate-solubilizing microbial strains is a valuable tool. The results regarding the solubilization of calcium phosphate in a hydroponic system with *Glycine max* plants highlighted the potential of *Lysinibacillus fusiformis* (PA26), which was the most efficient due to the increased P uptake and growth promotion observed in the inoculated plants. Gong et al. point out that root space availability and P supply significantly influence phosphorus use efficiency (PUE) in maize. However, the inoculation of mycorrhizal fungi allowed plants grown in pots with small diameters (20 cm) to increase total biomass even with reduced P supply. In a similar study, Gong et al. suggest that a higher P supply could compensate for the yield loss attributable to lower density; in addition, for the same P supply, the use of single superphosphate versus mono ammonium phosphate improved the growth of maize-grown at lower planting densities. To improve PUE and growth of maize and cucumber plants grown in hydroponics, Alzate Zuluaga et al. tested the efficacy of the 15S strain of *Enterobacter* sp. Inoculation of the bacterium under P-limiting conditions induced a greater effect on cucumber plants due to an increase in P allocation in both the epigeal and hypogean parts of the plants. In connection with bacterial gene expression, Yahya et al. studied the action of phosphate solubilization by *Ochrobactrum* sp. SSR (DSM 109610). A higher P content characterized wheat plants inoculated with DSM 109610; the bacterial supplementation increased the soil availability of this macronutrient and the alkaline phosphatase activity. The review work proposed by Rizvi et al. investigates the still under-studied role of the phosphate-soluble microbiome in managing sorghum cultivation-related diseases to reduce dependence on chemical pesticides further. In line with this, Haque et al. evaluated the biofertilising action of *Enterobacter* sp. HSTU-ASh6, a rice root endophyte that mineralizes chlorpyrifos. The results of tomatoes show improved growth of treated plants after foliar application of the HSTU-ASh6 strain, even with reduced nitrogen fertilization rates. In a pot experiment on maize, Chen et al. observed an increase in PUE and yield after the combined application of humic acids (45 kg ha^–1^) and an ammonium phosphate fertilizer compared to plants subjected to phosphorus fertilizer application alone. After extracting the humic acids (HA) in a phosphate fertilizer enhanced with HA, Jing et al. evaluated the effects on hydroponically grown maize seedlings. HA at doses of 2.5 and 5 mg C L^–1^ enhanced the average N and P uptake rates compared to untreated controls. To further investigate the effects of HA on plant growth, Wang et al. evaluated the effects of two different HAs [Aojia humic acid (AHA) and Shandong humic acid (SHA)] on maize as a model crop. After characterizing the humic acids through an ESI-HPLC-MS technology, the authors observed a different beneficial effect of AHA and SHA on maize growth (with AHA better than SHA) attributable to the significant differences in molecular compositions. Furthermore, in connection with HA, Xu et al. evaluated the effects of a root application (at 1%) on cucumber in three different hydroponic substrates (pure sand, pure cocopeat, and a mixture of sand and cocopeat). The results show that applying HA improved biometric parameters only in plants grown on pure cocopeat and cocopeat-sand mixture. Having identified, with mathematical models, the optimal doses of AMF and *Bacillus megaterium* (2.1 kg ha^–1^ for both), Ganugi et al. observed in co-inoculated maize plants an increased accumulation of N in the grain and an improved nitrogen harvest index. Through non-invasive 3D imaging of root architecture and specific biochemical assays, Mohammed et al. showed that root application of phosphite improved nitrogen assimilation and root biomass of maize seedlings. Chen et al. recorded an improvement in N, P, and K efficiency in sugarcane plants grown in China in two cultivation areas (Suixi and Wengyuan) following the application of algae extracts at different phenological states (sowing, early elongation, and early maturity). To reduce N application rates, Goñi et al. evaluated this macroelement’s uptake and assimilation mechanisms by plants treated with a biostimulant based on *Ascophyllum nodosum* extracts. The results on *Arabidopsis thaliana* showed a significant increase in nitrate content 6 days after application of the biostimulant. On the other hand, subsequent field trials revealed a significant increase in nitrate use efficiency on barley plants grown with 75% N supply. Similarly, Ciriello et al. demonstrated, on hydroponic basil, that applying a plant-derived protein hydrolysate (albeit dose-dependent) can compensate for the effects of a reduced nutrient solution (1 dS m^–1^), even improving yield and quality attributes. To improve the efficacy of synthetic fertilizers, Amaral and Brown investigated the effects of a plant-based inositol bioproduct on zinc (Zn) accumulation and mobility in wheat (*Triticum aestivum* L.) plants. Foliar application of the stimulant in combination with zinc sulfate significantly increased the concentration of this trace element in both grains and shoots compared to fertilized but not stimulated plants.

## 5 Biostimulants increase the qualitative characteristics of the produce

Interest in agri-food products that meet the goal of a healthy lifestyle has grown enormously in recent years. Various agronomic approaches have been proposed to enhance food products’ qualitative and functional attributes, including biostimulants (Ganugi et al., 2021; Melini et al., 2023). In this respect, Godlewska et al. observed after foliar application of plant extracts from common dandelion, valerian, and giant cabbage an improvement in vitamin C, macro- and micro-nutrient content, and a variable impact on the composition of fatty acids, and low molecular weight volatile compounds. Similarly, Abd-Elkader et al. evaluated the beneficial effects of *Trichoderma viride* and *Pseudomonas fluorescens* and three plant extracts of *Eucalyptus camaldulensis* (leaf extract indicated as LE), *Citrus sinensis* (leaf extract indicated as LE) and *Ficus benghalensis* fruits (fruit extract indicated as FE) with potassium silicate (K_2_SiO_3_) on the biochemical composition of courgette (*Cucurbita pepo* L.) fruits. Specifically, the LE combination with K_2_SiO_3_ increased greenhouse-grown zucchini fruits’ total phenolic content and antioxidant activity. Wang et al. evaluated the influence of five different concentrations of methyl jasmonate (MeJA) and salicylic acid (SA) on the yield and quality characteristics of Chinese chives. Applying MeJA and SA at concentrations of 500 and 150 μM resulted in a significant increase in phenols and flavonoids, vitamin C, and volatile compounds while reducing the nitrate content. Mesa et al., to evaluate the additive and/or synergistic effects of the application of a protein hydrolysate of animal origin combined with low-N priming, observed an improvement in quality parameters (soluble sugars, acidity, and lycopene content, vitamin E and vitamin C) on greenhouse tomatoes. Also, on tomatoes, Zhang et al. studied the effects of fulvic acids (obtained from maize straw) on fruit quality. The use of fulvic acids increased the concentrations of minerals (Ca, Fe, and Zn), citric acid, malic acid, and some amino acids without affecting the concentrations of soluble sugars. A field experiment of AMF inoculation on alfalfa (*Medicago sativa* L.) intended for livestock consumption increased the yield and nutrient and fatty acid content of the forage. Pellegrino et al. observed the persistence of positive effects even two years after inoculation due to an abundance of AMF at the root level. Although AMF-released glomalin’s functions are known, this molecule’s exogenous application is still unknown. Liu et al. investigated the effects of easily extractable glomalin-related soil protein (EE-GRSP) and difficultly extractable glomalin (DE-GRSP) on the growth and development of orange trifoliate (*Poncirus trifoliata* L.). The application of the two extracts resulted in contrasting effects. Specifically, the exogenous application of EE-GRSP enhanced growth, while a decrease was observed for plants treated with DE-GRSP. To improve the rooting of ‘Hurdal’ rose cuttings, Monder et al. tested different plant origin preparations (algae extracts, organic preparation, and plant extract). The best results were obtained using algae extracts for plant cuttings at the phenological stage of closed flower buds. Finally, Łangowski et al. observed on several soybean varieties cultivated in Canada and Brazil an increase in seed yield following the application of an *Ascophyllum nodosum* extract.

## 7 Conclusions and challenges ahead

It is an absolute prerogative that using plant biostimulants, irrespective of origin, will increasingly contribute to a mandatory transition to more economically and ecologically sustainable production systems. Agroecosystems that are more resilient and no longer threatened by uncontrolled human activities must be an integral part of future agriculture. Although the use of natural products and/or plant biostimulants is now a complementary tool to synthetic agrochemicals, the scientific community is called upon to define the underlying genetic, molecular, and physiological mechanisms, as a better understanding of these aspects would greatly facilitate the dissemination of bio-based products in the agricultural sector. Moreover, elucidating biostimulant × plant species × environment interaction × agricultural practices are crucial, in order to select optimal combinations in terms of yield, yield components and quality, and also optimizing application parameters (e.g. timing, mode, rate). Finally, it is also urgent to understand the interactions between biostimulant products, living organisms and chemical inputs (plant protection products and/or fertilizers) to develop more sustainable production systems.

## Author contributions

MR: Writing – review & editing, Writing – original draft. GC: Writing – review & editing, Writing – original draft. YR: Writing – review & editing, Writing – original draft.
