# Supplementary material for: Editorial: Biostimulants in agriculture II: towards a sustainable future
Source: Front Plant Sci. 2024 May 31;15:1427283. doi: 10.3389/fpls.2024.1427283 (PMC11176606; doi:10.3389/fpls.2024.1427283)
Supplement: Supplementary file 1 [file Table_1.docx]

**Supplementary Table 1.** Synopsis of published articles and topics

| **Title** | **Review or metanalysis and Perspective** | **Abiotic Stress** | **Biotic Stress** | **NUE** | **Quality traits** |  | **PH** | **SWE** | **HFA** | **PE** | **Silicon** | **Microorganisms** | **Compounds and ammendants** |  | **Cereals and industrial crops** | **Vegetables** | **Edible legumes** | **Tree and shrub crops** | **halophyte plants** |
| --- | --- | --- | --- | --- | --- | --- | --- | --- | --- | --- | --- | --- | --- | --- | --- | --- | --- | --- | --- |
| 2-Keto-L-Gulonic Acid Improved the Salt Stress Resistance of Non-heading Chinese Cabbage by Increasing L-Ascorbic Acid Accumulation |  | x |  |  |  |  |  |  |  |  |  |  | X |  |  | X |  |  |  |
| 5-Aminolevulinic Acid Improves Morphogenesis and Na^+^ Subcellular Distribution in the Apical Cells of Cucumis sativus L. Under Salinity Stress |  | x |  |  |  |  |  |  |  |  |  |  | X |  |  | X |  |  |  |
| Isolation and Evaluation of Rhizosphere *Actinomycetes* With Potential Application for Biocontrolling *Fusarium* Wilt of Banana Caused by *Fusarium oxysporum* f. sp. cubense Tropical Race |  |  | x |  |  |  |  |  |  |  |  |  |  |  |  |  |  | x |  |
| Food and agricultural wastes-derived biochars in combination with mineral fertilizer as sustainable soil amendments to enhance soil microbiological activity, nutrient cycling and crop production |  |  |  |  |  |  |  |  |  |  |  |  | X |  |  | X |  |  |  |
| Multiple Arbuscular Mycorrhizal Fungal Consortia Enhance Yield and Fatty Acids of *Medicago sativa*: A Two-Year Field Study on Agronomic Traits and Tracing of Fungal Persistence |  |  |  |  |  |  |  |  |  |  |  | X |  |  | X |  |  |  |  |
| AMF Inoculation Can Enhance Yield of Transgenic Bt Maize and Its Control Efficiency Against *Mythimna separata* Especially Under Elevated CO_2_ |  | x |  |  |  |  |  |  |  |  |  | X |  |  | X |  |  |  |  |
| Phosphorus-Use Efficiency Modified by Complementary Effects of P Supply Intensity With Limited Root Growth Space |  |  |  | x |  |  |  |  |  |  |  | X |  |  | X |  |  |  |  |
| Application of a Biostimulant (Pepton) Based in Enzymatic Hydrolyzed Animal Protein Combined With Low Nitrogen Priming Boosts Fruit Production Without Negatively Affecting Quality in Greenhouse-Grown Tomatoes |  |  |  |  | x |  | x |  |  |  |  |  |  |  |  | X |  |  |  |
| Evaluation of the Potential Use of a Collagen-Based Protein Hydrolysate as a Plant Multi-Stress Protectant |  | x |  |  |  |  | x |  |  |  |  |  |  |  | X |  |  |  |  |
| Identification and Characterization of *Bacillus tequilensis* GYUN-300: An Antagonistic Bacterium Against Red Pepper Anthracnose Caused by *Colletotrichum acutatum* in Korea |  |  | x |  |  |  |  |  |  |  |  | X |  |  |  | X |  |  |  |
| Evaluation and Genome Analysis of *Bacillus subtilis* YB-04 as a Potential Biocontrol Agent Against *Fusarium* Wilt and Growth Promotion Agent of Cucumber |  |  | x |  |  |  |  |  |  |  |  | X |  |  |  | X |  |  |  |
| Physiological Biochemistry-Combined Transcriptomic Analysis Reveals Mechanism of Bacillus cereus G2 Improved Salt-Stress Tolerance of *Glycyrrhiza uralensis* Fisch. Seedlings by Balancing Carbohydrate Metabolism |  | x |  |  |  |  |  |  |  |  |  | X |  |  | X |  |  |  |  |
| Can Inoculation With the Bacterial Biostimulant *Enterobacter* sp. Strain 15S Be an Approach for the Smarter P Fertilization of Maize and Cucumber Plants? |  |  |  | x |  |  |  |  |  |  |  | X |  |  | X | X |  |  |  |
| Seed Biopriming With *Trichoderma* Strains Isolated From Tree Bark Improves Plant Growth, Antioxidative Defense System in Rice and Enhance Straw Degradation Capacity |  | x | x |  |  |  |  |  |  |  |  | X |  |  | X |  |  |  |  |
| Biostimulants induce positive changes in the radish morpho-physiology and yield |  |  |  |  |  |  |  |  |  | x |  |  | X |  |  | X |  |  |  |
| Co-composted Biochar Enhances Growth, Physiological, and Phytostabilization Efficiency of *Brassica napus* and Reduces Associated Health Risks Under Chromium Stress |  | x |  |  |  |  |  |  |  |  |  |  | X |  | X |  |  |  |  |
| Myco-Synergism Boosts Herbivory-Induced Maize Defense by Triggering Antioxidants and Phytohormone Signaling |  |  | x |  |  |  |  |  |  |  |  | X | X |  | X |  |  |  |  |
| Coated Diammonium Phosphate Combined With Humic Acid Improves Soil Phosphorus Availability and Photosynthesis and the Yield of Maize |  |  |  | x |  |  |  |  | x |  |  |  | X |  | X |  |  |  |  |
| Assessment of digestates prepared from maize, legumes, and their mixed culture as soil amendments: Effects on plant biomass and soil properties |  |  |  |  |  |  |  |  |  |  |  |  | X |  | X |  | x |  |  |
| Dose-Dependent Application of Straw-Derived Fulvic Acid on Yield and Quality of Tomato Plants Grown in a Greenhouse |  |  |  |  | x |  |  |  | x |  |  |  |  |  |  | X |  |  |  |
| Endophytic Bacterial Isolates From Halophytes Demonstrate Phytopathogen Biocontrol and Plant Growth Promotion Under High Salinity |  | x | x |  |  |  |  |  |  |  |  | X |  |  |  |  |  |  | x |
| Endophytic *Bacillus altitudinis* Strain Uses Different Novelty Molecular Pathways to Enhance Plant Growth |  |  |  |  |  |  |  |  |  |  |  | X |  |  | X |  |  |  |  |
| Unveiling chlorpyrifos mineralizing and tomato plant-growth activities of *Enterobacter* sp. strain HSTU-ASh6 using biochemical tests, field experiments, genomics, and in silico analyses |  |  |  | x |  |  |  |  |  |  |  | X |  |  |  | X |  |  |  |
| Adaptive Reprogramming During Early Seed Germination Requires Temporarily Enhanced Fermentation-A Critical Role for Alternative Oxidase Regulation That Concerns Also Microbiota Effectiveness | X |  |  |  |  |  |  |  |  |  |  | X |  |  |  |  |  |  |  |
| Differential Effects of Exogenous Glomalin-Related Soil Proteins on Plant Growth of Trifoliate Orange Through Regulating Auxin Changes |  |  |  |  |  |  |  |  |  |  |  | X | X |  |  |  |  | x |  |
| Humic Acid Modified by Being Incorporated Into Phosphate Fertilizer Increases Its Potency in Stimulating Maize Growth and Nutrient Absorption |  |  |  | x |  |  |  |  | x |  |  |  |  |  | X |  |  |  |  |
| Effect of Humic Acid Addition on Buffering Capacity and Nutrient Storage Capacity of Soilless Substrates |  |  |  | x |  |  |  |  | x |  |  |  |  |  |  | X |  |  |  |
| Analysis of the molecular composition of humic substances and their effects on physiological metabolism in maize based on untargeted metabolomics |  |  |  |  |  |  |  |  | x |  |  |  |  |  | X |  |  |  |  |
| Foliar Application of an Inositol-Based Plant Biostimulant Boosts Zinc Accumulation in Wheat Grains: A µ-X-Ray Fluorescence Case Study |  |  |  |  |  |  |  |  |  |  |  |  | X |  | X |  |  |  |  |
| Integration of Phenomics and Metabolomics Datasets Reveals Different Mode of Action of Biostimulants Based on Protein Hydrolysates in *Lactuca sativa* L. and *Solanum lycopersicum* L. Under Salinity |  | x |  |  |  |  | x |  |  |  |  |  |  |  |  | X |  |  |  |
| Liquiritoside Alleviated Pb Induced Stress in *Brassica rapa* subsp. Parachinensis: Modulations in Glucosinolate Content and Some Physiochemical Attributes |  | x |  |  |  |  |  |  |  |  |  |  | X |  |  | X |  |  |  |
| Habitat-adapted heterologous symbiont *Salinispora arenicola* promotes growth and alleviates salt stress in tomato crop plants |  | x |  |  |  |  |  |  |  |  |  | X |  |  |  | X |  |  |  |
| Melatonin Enhances Drought Tolerance by Regulating Leaf Stomatal Behavior, Carbon and Nitrogen Metabolism, and Related Gene Expression in Maize Plants |  | x |  |  |  |  |  |  |  |  |  |  | X |  | X |  |  |  |  |
| Optimization of the growth conditions through response surface methodology and metabolomics for maximizing the auxin production by *Pantoea agglomerans* C1 |  |  |  |  |  |  |  |  |  |  |  | X |  |  |  |  |  |  |  |
| Effects of Preharvest Methyl Jasmonate and Salicylic Acid Treatments on Growth, Quality, Volatile Components, and Antioxidant Systems of Chinese Chives |  |  |  |  | x |  |  |  |  |  |  |  | X |  |  | X |  |  |  |
| Root Reinforcement Improved Performance, Productivity, and Grain Bioactive Quality of Field-Droughted Quinoa (*Chenopodium quinoa*) |  | x |  |  | x |  |  |  |  |  |  | X | X |  | X |  |  |  |  |
| Efficiency of the Hydroponic System as an Approach to Confirm the Solubilization of CaHPO_4_ by Microbial Strains Using *Glycine max* as a Model |  |  |  | x |  |  |  |  |  |  |  | X |  |  | X |  |  |  |  |
| The Use of Interactions Between Microorganisms in Strawberry Cultivation (*Fragaria x ananassa* Duch.) |  |  | x |  |  |  |  |  |  |  |  | X |  |  |  | X |  |  |  |
| A response surface methodology approach to improve nitrogen use efficiency in maize by an optimal mycorrhiza-to-*Bacillus* co-inoculation rate |  |  |  | x |  |  |  |  |  |  |  | X |  |  | X |  |  |  |  |
| Limiting-Stress-Elimination Hypothesis: Using Non-hormonal Biostimulant to Reduce Stress and Increase Savanna Cowpea [*Vigna unguiculata* (L.) Walp.] Productivity |  | x |  |  |  |  |  |  |  |  |  |  | X |  |  |  | x |  |  |
| Photosynthetic Pigments and Biochemical Response of Zucchini (*Cucurbita pepo* L.) to Plant-Derived Extracts, Microbial, and Potassium Silicate as Biostimulants Under Greenhouse Conditions |  |  |  |  | x |  |  |  |  | x | x | X |  |  |  | X |  |  |  |
| *Pseudomonas palmensis* sp. nov., a Novel Bacterium Isolated From *Nicotiana glauca* Microbiome: Draft Genome Analysis and Biological Potential for Agriculture |  |  |  |  |  |  |  |  |  |  |  | X |  |  |  | X |  | x |  |
| A novel PGPF *Penicillium olsonii* isolated from the rhizosphere of *Aeluropus littoralis* promotes plant growth, enhances salt stress tolerance, and reduces chemical fertilizers inputs in hydroponic system |  | x |  | x |  |  |  |  |  |  |  | X |  |  | X |  |  |  |  |
| *Lactobacillus helveticus* EL2006H cell-free supernatant enhances growth variables in *Zea mays* (maize), *Glycine max* L. Merill (soybean) and *Solanum tuberosum* (potato) exposed to NaCl stress |  | x |  |  |  |  |  |  |  |  |  | X |  |  | X |  |  |  |  |
| The stimulatory effect of Thuricin 17, a PGPR-produced bacteriocin, on canola (*Brassica, napus* L.) germination and vegetative growth under stressful temperatures |  | x |  |  |  |  |  |  |  |  |  | X |  |  | X |  |  |  |  |
| Impact of Plant Growth-Promoting Rhizobacteria Inoculation and Grafting on Tolerance of Tomato to Combined Water and Nutrient Stress Assessed via Metabolomics Analysis |  | x |  |  |  |  |  |  |  |  |  | X |  |  |  | X |  |  |  |
| *Rahnella aquatilis* JZ-GX1 alleviates iron deficiency chlorosis in *Cinnamomum camphora* by secreting desferrioxamine and reshaping the soil fungal community |  | x |  |  |  |  |  |  |  |  |  | X |  |  |  |  |  | x |  |
| Comparative Transcriptomics and Metabolomics Reveal an Intricate Priming Mechanism Involved in PGPR-Mediated Salt Tolerance in Tomato |  | x |  |  |  |  |  |  |  |  |  | X |  |  |  | X |  |  |  |
| Biostimulatory Action of Vegetal Protein Hydrolysate Compensates for Reduced Strength Nutrient Supply in a Floating Raft System by Enhancing Performance and Qualitative Features of “Genovese” Basil |  |  |  | x | x |  | x |  |  |  |  |  |  |  |  | X |  |  |  |
| Different vegetal protein hydrolysates distinctively alleviate salinity stress in vegetable crops: A case study on tomato and lettuce |  | x |  |  |  |  | x |  |  |  |  |  |  |  |  | X |  |  |  |
| Integration of Gas Exchange With Metabolomics: High-Throughput Phenotyping Methods for Screening Biostimulant-Elicited Beneficial Responses to Short-Term Water Deficit |  | x |  |  |  |  |  |  |  |  |  |  | X |  |  | X |  |  |  |
| Designing Synergistic Biostimulants Formulation Containing Autochthonous Phosphate-Solubilizing Bacteria for Sustainable Wheat Production |  |  |  | x |  |  |  |  | x |  |  | X | X |  | X |  |  |  |  |
| Phosphite treatment can improve root biomass and nutrition use efficiency in wheat |  |  |  | x |  |  |  |  |  |  |  |  | X |  | X |  |  |  |  |
| Complementary effects of phosphorus supply and planting density on maize growth and phosphorus use efficiency |  |  |  | x |  |  |  |  |  |  |  |  |  |  | X |  |  |  |  |
| Systematic Investigation of the Effects of Seven Plant Extracts on the Physiological Parameters, Yield, and Nutritional Quality of Radish (*Raphanus sativus* var. sativus) |  |  |  |  | x |  |  |  |  | x |  |  |  |  |  | X |  |  |  |
| Potential of Algae–Bacteria Synergistic Effects on Vegetable Production | X |  |  |  |  |  |  |  |  |  |  | X | X |  |  | X |  |  |  |
| Contribution of Arbuscular Mycorrhizal Fungi, Phosphate–Solubilizing Bacteria, and Silicon to P Uptake by Plant | X |  |  | x |  |  |  |  |  |  | x | X |  |  |  |  |  |  |  |
| Ericoid mycorrhizal fungi as biostimulants for improving propagation and production of ericaceous plants | X | x | x |  |  |  |  |  |  |  |  | X |  |  |  |  |  |  |  |
| Application of biostimulant products and biological control agents in sustainable viticulture: A review | X |  |  |  |  |  | x | x | x |  |  | X |  |  |  |  |  | x |  |
| Perspectives and potential applications of endophytic microorganisms in cultivation of medicinal and aromatic plants | X | x | x |  |  |  |  |  |  |  |  | X |  |  | X | X |  |  |  |
| A Global Network Meta-Analysis of the Promotion of Crop Growth, Yield, and Quality by Bioeffectors | X |  |  | x | x |  | x | x | x |  |  |  |  |  |  |  |  |  |  |
| A Meta-Analysis of Biostimulant Yield Effectiveness in Field Trials | X | x |  | x |  |  | x | x | x | x | x | X |  |  | X | X |  | x |  |
| A Combined Use of Rhizobacteria and Moringa Leaf Extract Mitigates the Adverse Effects of Drought Stress in Wheat (*Triticum aestivum* L.) |  | x |  |  |  |  |  |  |  | x |  | X |  |  | X |  |  |  |  |
| Bioformulation of Silk-Based Coating to Preserve and Deliver *Rhizobium tropici* to *Phaseolus vulgaris* Under Saline Environments |  | x |  |  |  |  |  |  |  |  |  |  | X |  |  |  | x |  |  |
| The Role of Plant Origin Preparations and Phenological Stage in Anatomy Structure Changes in the Rhizogenesis of Rosa “Hurdal” |  |  |  |  |  |  |  | x |  | x |  |  | X |  |  |  |  | x |  |
| Is foliar spectrum predictive of belowground bacterial diversity? A case study in a peach orchard |  |  |  |  |  |  |  |  |  |  |  | X |  |  |  |  |  | x |  |
| Biostimulant Capacity of an Enzymatic Extract From Rice Bran Against Ozone-Induced Damage in *Capsicum annum* |  | x |  |  |  |  |  |  |  | x |  |  |  |  |  | X |  |  |  |
| Iron Oxide and Silicon Nanoparticles Modulate Mineral Nutrient Homeostasis and Metabolism in Cadmium-Stressed *Phaseolus vulgaris* |  | x |  |  |  |  |  |  |  |  | x |  | X |  | X |  |  |  |  |
| Silicon-Mediated Priming Induces Acclimation to Mild Water-Deficit Stress by Altering Physio-Biochemical Attributes in Wheat Plants |  | x |  |  |  |  |  |  |  |  | x |  |  |  | X |  |  |  |  |
| Addressing the contribution of small molecule-based biostimulants to the biofortification of maize in a water restriction scenario |  | x |  |  | x |  |  |  |  |  |  |  | X |  | X |  |  |  |  |
| Sorghum-Phosphate Solubilizers Interactions: Crop Nutrition, Biotic Stress Alleviation, and Yield Optimization | X |  |  | x | x |  |  |  |  |  |  | X |  |  | X |  |  |  |  |
| Comparative study of the chemical composition and antifungal activity of commercial brown seaweed extracts |  |  | x |  |  |  |  | x |  |  |  |  |  |  |  |  |  |  |  |
| Reducing Nitrogen Input in Barley Crops While Maintaining Yields Using an Engineered Biostimulant Derived From *Ascophyllum nodosum* to Enhance Nitrogen Use Efficiency |  |  |  | x |  |  |  | x |  |  |  |  |  |  | X |  |  |  |  |
| *Ascophyllum nodosum* Extract (Sealicit^TM^) Boosts Soybean Yield Through Reduction of Pod Shattering-Related Seed Loss and Enhanced Seed Production |  |  |  |  |  |  |  | x |  |  |  |  |  |  | X |  |  |  |  |
| Effects of Seaweed Extracts on the Growth, Physiological Activity, Cane Yield and Sucrose Content of Sugarcane in China |  |  |  |  |  |  |  | x |  |  |  |  |  |  | X |  |  |  |  |
| Agronomic efficiency and genome mining analysis of the wheat-biostimulant rhizospheric bacterium *Pseudomonas pergaminensis* sp. nov. strain 1008^T^ |  |  |  |  |  |  |  |  |  |  |  | X |  |  | x |  |  |  |  |
